# Three-dimensional depth sensor imaging to identify adolescent idiopathic scoliosis: a prospective multicenter cohort study

**DOI:** 10.1038/s41598-019-46246-0

**Published:** 2019-07-04

**Authors:** Terufumi Kokabu, Noriaki Kawakami, Koki Uno, Toshiaki Kotani, Teppei Suzuki, Yuichiro Abe, Kenichiro Maeda, Fujio Inage, Yoichi M. Ito, Norimasa Iwasaki, Hideki Sudo

**Affiliations:** 1 Department of Orthopaedic Surgery, Hokkaido University Hospital, N14W5, Sapporo, Hokkaido 060-8648 Japan; 2 Department of Orthopaedic Surgery, Eniwa Hospital, Koganechuo 2-1-1, Eniwa, Hokkaido 061-1449 Japan; 3 Department of Orthopaedic Surgery, Meijo Hospital, Sannomal 1-3-1, Nagoya, Aichi 460-0001 Japan; 4 Department of Orthopaedic Surgery, National Hospital Organization, Kobe Medical Center, Nishiochiai 3-1-1, Kobe, Hyogo 654-0155 Japan; 5 Department of Orthopaedic Surgery, Seirei Sakura Citizen Hospital, Eharadai 2-36-2, Sakura, Chiba 285-8765 Japan; 6 Clinical Research and Medical Innovation Center, Hokkaido University Hospital, N14W5, Sapporo, Hokkaido 060-8648 Japan; 7 Department of Biostatistics, Faculty of Medicine and Graduate of Medicine, Hokkaido University, N15W7, Sapporo, Hokkaido 060-8638 Japan; 8 Department of Advanced Medicine for Spine and Spinal Cord Disorders, Faculty of Medicine and Graduate of Medicine, Hokkaido University, N15W7, Sapporo, Hokkaido 060-8638 Japan

**Keywords:** Adaptive clinical trial, Orthopaedics, Diagnosis

## Abstract

Adolescent idiopathic scoliosis is the most ordinary pediatric spinal disease that causes a three-dimensional deformity. Early detection of this potentially progressive deformity is considered crucial. The purpose of the present study was to report the potential for accurately diagnosis of adolescent idiopathic scoliosis using a newly developed, automated, noninvasive asymmetry-recognition system for the surface of the human back using a three-dimensional depth sensor. We included 170 subjects with suspected adolescent idiopathic scoliosis in this study. Outcomes measured included patient demographics, Cobbe angles from radiographic measurements, and asymmetry indexes. The coefficient of correlation between the asymmetry index and the Cobb angle was 0.85. For the prediction of scoliosis >10°, the area under the curve was 0.98, sensitivity was 0.97, specificity was 0.93, positive predictive value was 0.99, negative predictive value was 0.72, accuracy was 0.97, positive likelihood ratio was 13.55, and negative likelihood ratio was 0.04. The posterior test probability for the positive screen >10° was 98.9% if the asymmetry index was >1.268, three times in a row. This novel system automatically evaluated the back asymmetry. Therefore, this study demonstrates the outstanding discriminative ability of this newly developed system for deciding whether an examinee should undergo additional radiography to define scoliosis. This system can be used as an alternative to the forward bend test and scoliometer measurement in clinics. Future studies should seek to confirm these findings in a larger group and involve mass school scoliosis screening programs within the context of a multicenter trial.

## Introduction

Adolescent idiopathic scoliosis (AIS) is the most ordinary pediatric spinal disease and is traditionally defined as a lateral curvature of the spine equal to or >10° in persons aged 10–18 years that is not a result of an underlying condition^1^. The onset of AIS typically concurs with growth spurt. Moderate scoliosis (25–40°) is treated nonsurgically, using a brace to avoid further advancement during periods of growth. Patients with severe AIS (>40–50°) can be considered for surgery if they are not responsive to non-operative modalities^2–5^.

Early detection of this potentially progressive deformity is considered crucial for intervention. Frequent observation is needed to confirm if curvatures are aggravated during growing periods^2^. Several methods can be applied to screen for AIS. The most basic of these is Adam’s forward bend test with the use of a scoliometer^6–8^. Although this examination is highly sensitive (83.3%) and specific (86.8%)^6,7^, the correlation between scoliometer values and Cobb angles is unsatisfactory (*r* = 0.677)^8^. Furthermore, the examiner must determine where the scoliometer should be placed and manually obtain all measurements. This is problematic for doctors or school nurses who carefully observe many subjects within a limited time frame^2^. Moiré topography utilizes a specialized device that projects contour lines onto a person’s back. This modality requires that the light-source direction be perpendicular to the back surface^2^, which is not possible when the trunk is bent forward, resulting in false positive rates of 32–60%^9–12^. Thus, this screening modality is used infrequently^1^.

Recently, we created a new system that addresses the problems associated with AIS identification^2^. The system can measure the degree of asymmetry on the surface of the human back with three-dimensional (D) measurements generated by a consumer-grade 3D depth sensor and a laptop computer with the algorithm installed. This enables automated evaluation of back asymmetry as an asymmetry index^2^. The pilot method validation study^2^ (n = 76) showed that the average time from capture to analysis was 1.5 seconds and the coefficient of correlation between the asymmetry index and the Cobb angle was 0.88. Body Mass Index had a minor effect on the asymmetry index. When predicting AIS of >20°, the area under the curve was 0.94, sensitivity was 0.89, and specificity was 0.91.

We also evaluated intra-observer repeatability using the subjects who assented to the test^2^. This cohort included healthy subjects, and the intraclass correlation coefficient was 0.955, indicating excellent repeatability^2^. These results suggested the discriminative ability for deciding whether an examinee needed additional radiography to definitively diagnose scoliosis^2^.

Due to the onset age of AIS, screening efforts are focused on school-aged adolescents and are considered important for early detection, although such nationwide strategies were previously considered implausible due to the high associated cost and lack of skilled operators. As a necessary next step in the progression of this novel 3D asymmetry evaluation system, and to determine its potential for use in clinics or during physical examinations at schools, we sought to report the outcomes of a prospective, multicenter, cohort study.

## Methods

### Identification of cases

The study was conducted in five scoliosis centers in Japan between June 2018 and December 2018. Hokkaido University served as the data-coordinating center for the study and was responsible for entering data on all subjects. Institutional reviews board approval was obtained from all participating centers (Institutional review board of Hokkaido University Hospital, Institutional review board of Eniwa Hospital, Institutional review board of Meijo Hospital, Institutional review board of Kobe Medical Center, and Institutional review board of Seirei Sakura Citizen Hospital). All methods were performed in accordance with the relevant guidelines and regulations. An independent organization audited and monitored this study.

Subjects were referred to our hospitals on suspicion of AIS. The inclusion criteria were as follows^2^: (i) age 7–18 years; (ii) referred for confirmed diagnosis based on radiography^2^; (iii) no history of brace treatment; (iv) willingness and ability to provide written informed consent and/or informed assent. The informed consent or assent form was obtained from high school students (age 16–18 years old) or elementary/junior high school students (age 7–15 years old), respectively. In addition, the informed consent form was obtained from all guardians. We selected these age ranges because the School Health and Safety Act in Japan requires scoliosis screenings beginning in elementary school (7 years old) to high school (18 years old), although common practice guidelines recommend screening girls at 10 and 12 years and boys at 13 years age. Exclusion criteria were syndromic, neuromuscular, and congenital scoliosis. We explained the procedure, indications, and preparation and participants indicated their understanding and obtained written informed consent^2^. Following consent, we accessed the subjects’ medical records, where we obtained information pertaining to age, sex, and Cobb angle measured from digital radiographs. Cobb angles were measured three times by only one spine specialist and the averages were used.

### Three-dimensional depth sensor imaging

The system consists of a consumer-grade 3D depth sensor (Xtion Pro Live, ASUSTeK Computer Inc. Taipei, Republic of China) and a laptop computer (Core-i5, 7200U-4 GB HP pavilion-15-au105tu, HP Inc, California, United States)^2^. The system was located in our orthopaedic offices and can be instantly utilized following installation of the external 3D deep sensor, which can be set up within a few minutes^2^. Additionally, the sensor requires only a single calibration^2^.

Our fully automated algorithm for detecting 3D asymmetry has been previously reported in detail^2^. This algorithm included the following procedures:

#### Recognition of point clouds from the body surface

The surface of the patient’s back is scanned by the depth sensor so that the transverse lines of right and left of the waist are roughly aligned with the recommended lines displayed on the computer monitor. A 3D point cloud P1 is captured.

#### Extraction of approximated median sagittal plane and region of interest

**Figure 1:**
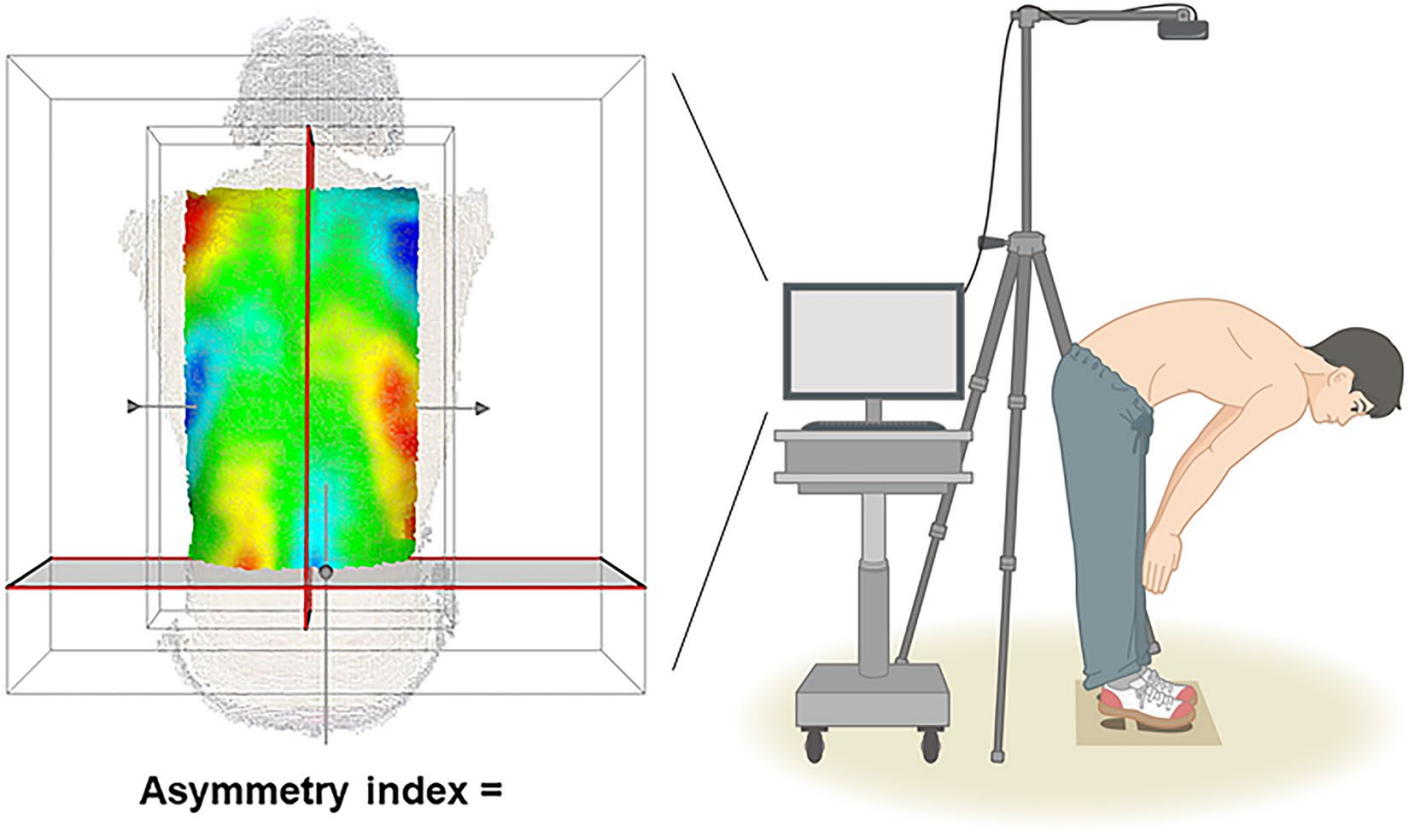
Detection of three-dimensional asymmetries. The subject bends forward, and the surface of the back is scanned by a 3D depth sensor. There was a template to constraint positioning that illustrated feet as parallel position. We extract an approximated median sagittal plane and region of interest, which is defined as a rectangle box generated using the patient’s waistlines and shoulders as the four corners of the box.

The region of interest is defined as a rectangular box generated from the waist on both sides of the body to both shoulders (Fig. 1). Principal component analysis is applied to the point cloud P1, and the pose-normalized point cloud P2 is obtained from the P1 where the approximate median sagittal plane is aligned.

#### Generation of reflected point clouds

To carry out the asymmetry analysis, a reflected point cloud P2r is first generated by taking a mirror projection of P2 based on the sagittal plane.

#### Determine the best fit between original point clouds and reflected point clouds

We use an iterative closest point method to obtain an optimum position and orientation for P2r best fitted to P2.

#### Calculation of the asymmetry index

The difference in the position of P2r relative to P2 is evaluated, and the difference distribution is rendered as a color map. In addition, the asymmetry index is calculated based on the average deviation between the original point clouds and the corresponding best fit and reflected point clouds.

After subjects removed their upper clothing, they were subjected to the Adam’s forward bend test^6,7^ followed by shooting with the sensor^2^. To avoid cases in which the patient’s posture obviously deviated from the recommended posture, such as cases in which the subject’s back is not visible in the monitor, the system guides the following countermeasures^2^: (1) The position in which subjects should place their feet are marked^2^. There was a template to constraint positioning that illustrated feet as parallel position (Fig. 1)^2^. In addition, a rectangular box that designates the recommended positioning is displayed on the monitor during photography^2^. The subject can be positioned based on this frame and visually guided to approach the advocated position in the x (left–right) and y (craniocaudal) directions, and attain the appropriate z rotation angle^2^. (2) The angular difference between the approximate plane of the 3D point cloud in the detection region of the photographed subject, and the direction of photography of the 3D sensor is calculated during photography^2^. This value can be evaluated to determine if it is within the threshold value; via analysis of the 3D data, the rotation angles along the x direction (<±7.5°), and the angular rotation along the y direction (<±15°) can be made to approach the recommended state^2^. A female medical secretary used the system. She did not need special training for this system. The average time from scanning to result was 1.5 s^2^.

### Statistical analysis

Sample sizes for the quantitative data were determined as follows. Our previous pilot study showed that the coefficient correlation between the asymmetry index and the Cobb angle was 0.88^2^. It has been reported that the correlation coefficient between the scoliometer value and the Cobb angle was 0.677^8^. We expected that the correlation coefficient of the asymmetry index in the present study would exceed the scoliometer value and be >0.7. We further expected that the correlation coefficient in this study would be >0.8 and that the type II error rate, beta, would be 0.2. Using a Fisher r-to-z transformation the required sample size was 150. To account for potential cases of minor scanning error, we finally determined the sample size as 170.

Pearson’s correlation coefficient analyses were applied to evaluate relationships between the Cobb angle and the asymmetry index^2^. A Fisher r-to-z transformation was applied to test for the difference between correlation coefficients relative to sex or curve types^2^. Receiver operating characteristic (ROC) analyses were applied to estimate the best cut-off values for the asymmetry index and to predict Cobb angles >10°, 15°, 20°, or 25°^2^.

The performance of the asymmetry indices was assessed using the area under the ROC curve (AUC)^2^. AUCs were categorized as follows^2^: no discrimination (AUC = 0.50); acceptable discrimination (0.7 ≤ AUC < 0.8); excellent discrimination (0.8 ≤ AUC < 0.9); and outstanding discrimination (AUC ≥ 0.9)^13^. To determine the cut-off values, we applied the Youden index, which is computed as sensitivity + specificity − 1, for each potential cut-off point; the optimal cut-off point is the tool score with the highest value^2,14^. Based on the cut-off values, the sensitivities, specificities, positive predictive values, negative predictive values, accuracy, positive likelihood ratios, and negative likelihood ratios were determined^2^.

Bayes’ theorem was used to determine the posterior test probabilities of a scoliosis-positive or -negative screen using cut-off values for the asymmetry index. The posterior test probabilities (Ppost) were calculated using the prior test probabilities (Ppri) and likelihood ratios (LR) using the following equation:

$${\rm{Ppost}}/(1-{\rm{Ppost}})={\rm{LR}}\,\ast \,{\rm{Ppri}}/(1-{\rm{Ppri}})$$

Because the prevalence of AIS >10° was reported to be 3.5%^15^, this prevalence value was used as the prior test probabilities.

Data analyses were performed using JMP statistical software for Windows (version 12; SAS, Inc., Cary, NC, USA)^2^. *P* < 0.05 was considered statistically significant.

## Results

**Figure 2:**
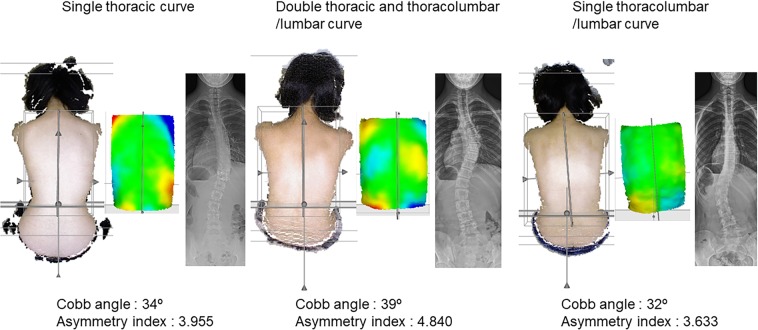
Visual results for each type of curves.

We obtained data from 170 subjects (21 males and 149 females). The mean age was 14.3 years (range, 8 to 18 years). The average Cobb angle was 25.2° (range, 0° to 60.7°). When subjects with a Cobb angle of 0° were excluded^2^, the average Cobb angle of single thoracic curve (n = 70), double thoracic and thoracolumbar/lumbar curve (n = 47), and single thoracolumbar/lumbar (n = 49) were 24.9° (range, 3.1° to 59.5°), 31.6° (range, 13.5° to 60.7°), and 20.7° (range, 6.7° to 54.4°), respectively. Visual results for each type of curves were also presented as Fig. 2. The mean asymmetry index was 3.086 (range, 0.737 to 8.699).

**Figure 3:**
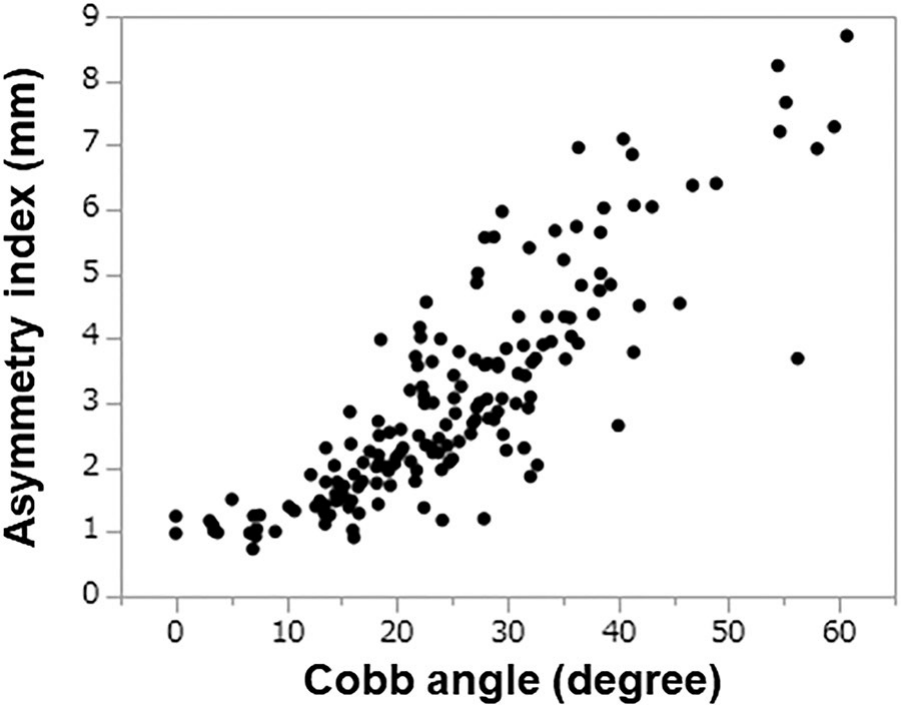
Correlation between the asymmetry index and Cobb angle.

For qualitative evaluation, the asymmetry index was compared with the Cobb angle^2^. There was no significant difference between the correlation coefficient in males (n = 21, r = 0.89) and females (n = 149, r = 0.85) (p = 0.42) using the Fisher r-to-z transformation. Because there was no difference, genders were combined for further analysis. The Pearson’s correlation coefficient between the Cobb angle and the asymmetry index was 0.85 (n = 170, p < 0.01) (Fig. 3). When subjects with a Cobb angle of 0° were excluded^2^, there was no significant difference between the correlation coefficients for single thoracic curve (n = 70, r = 0.87), double thoracic and thoracolumbar/lumbar curve (n = 47, r = 0.81), and single thoracolumbar/lumbar curve (n = 49, r = 0.88) (p = 0.40) using the Fisher r-to-z transformation.

### Scoliosis predictions

**Figure 4:**
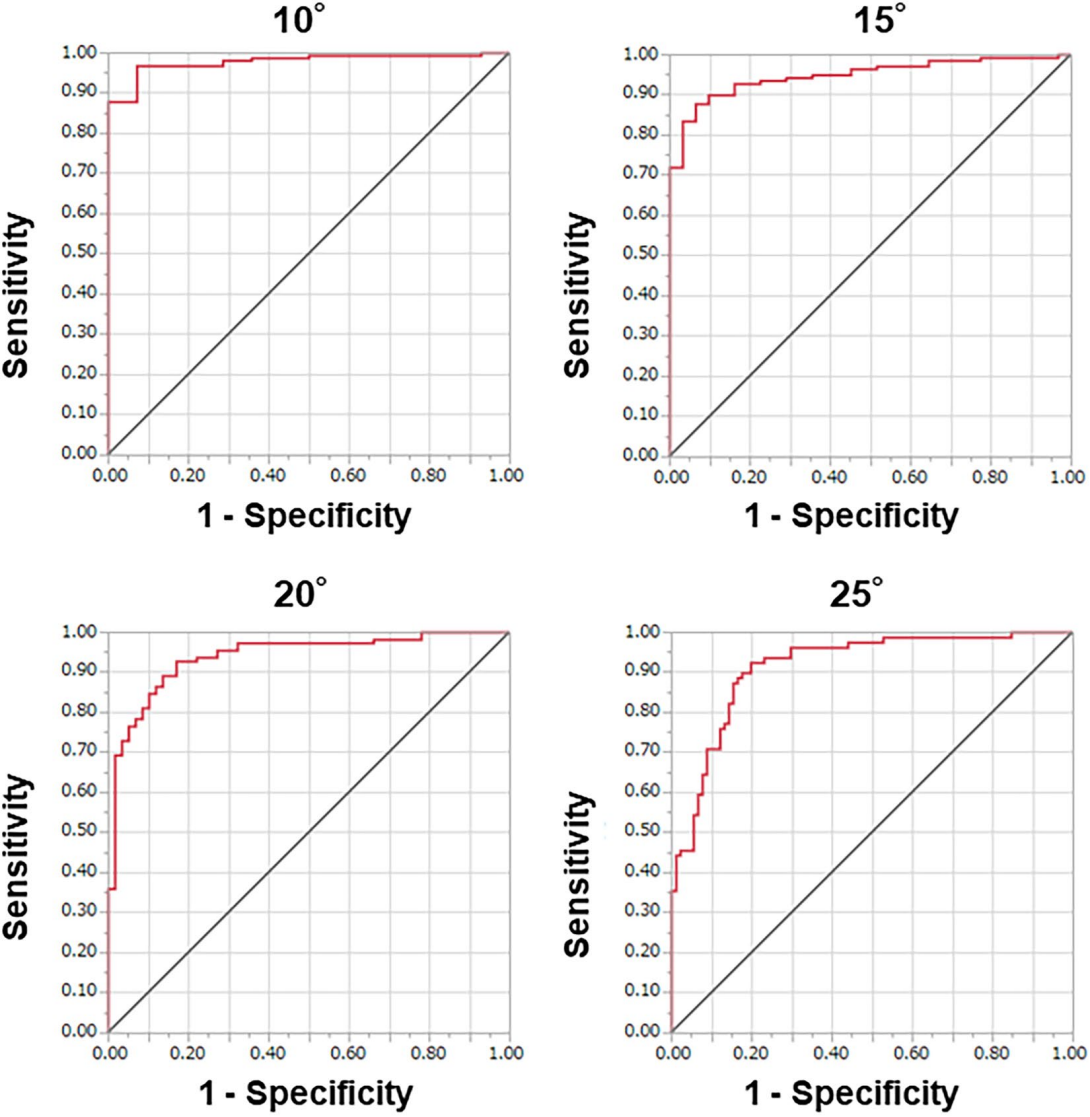
Receiver operating characteristic curves plotted using the asymmetry index.

We assessed the ability of this system to predict AIS (Table 1). The ROC curves were plotted using the asymmetry index (Fig. 4) and revealed that the cut-off value of the asymmetry index was 1.268 when a Cobb angle of >10° was selected for patients diagnosed with AIS. When a Cobb angle >10° was predicted, the AUC was 0.98, sensitivity was 0.97, specificity was 0.93, positive predictive value was 0.99, negative predictive value was 0.72, accuracy was 0.97, positive likelihood ratio was 13.55, and negative likelihood ratio was 0.04. The AUC had outstanding discrimination ability. Results based on Cobb angles of 15°, 20°, and 25° are additionally shown in Fig. 4 and Table 1. ROC analysis results, with breakdown by curve type, are shown in Table 1. In addition, the posterior test probability results are shown in Table 2. The posterior test probability for a positive screen >10° was 98.9% if the asymmetry index was >1.268 three times in a row.

**Table 1:** ROC analysis with Cobb angle.

Curve type	Cobb angle	Cut-off value	AUC (95% CI)	Sensitivity	Specificity	PPV	NPV	Accuracy	PLR	NLR
Total subjects (n = 170)	10°	1.268	0.98 (0.94, 0.99)	0.97	0.93	0.99	0.72	0.97	13.55	0.04
	15°	1.893	0.95 (0.91, 0.97)	0.88	0.94	0.98	0.63	0.89	13.60	0.13
	20°	2.081	0.94 (0.89, 0.97)	0.93	0.83	0.91	0.86	0.89	5.48	0.09
	25°	2.514	0.92 (0.86, 0.95)	0.92	0.80	0.80	0.92	0.86	4.67	0.10
Single thoracic curve (n = 70)	10°	1.568	0.98 (0.90, 1.00)	0.97	1.00	1.00	0.78	0.97	∞	0.03
	15°	1.893	0.97 (0.90, 0.99)	0.92	1.00	1.00	0.67	0.93	∞	0.08
	20°	2.231	0.94 (0.84, 0.98)	0.96	0.78	0.90	0.90	0.90	4.40	0.05
	25°	2.514	0.91 (0.81, 0.96)	0.97	0.74	0.79	0.96	0.86	3.78	0.04
Double thoracic and thoracolumbar/lumbar curve (n = 47)	10°	−	−	−	−	−	−	−	−	−
	15°	1.862	0.96 (0.96, 0.96)	0.96	1.00	1.00	0.33	0.96	∞	0.04
	20°	2.746	0.88 (0.73, 0.95)	0.73	1.00	1.00	0.35	0.77	∞	0.27
	25°	2.746	0.84 (0.69, 0.93)	0.84	0.80	0.90	0.71	0.83	4.22	0.20
Single thoracolumbar/lumbar curve (n = 49)	10°	1.268	0.98 (0.89, 1.00)	0.93	1.00	1.00	0.63	0.94	∞	0.07
	15°	1.787	0.87 (0.74, 0.94)	0.77	0.89	0.92	0.70	0.82	6.97	0.25
	20°	2.081	0.93 (0.78, 0.98)	0.91	0.93	0.91	0.93	0.92	12.27	0.10
	25°	3.430	0.96 (0.87, 0.99)	0.91	0.95	0.83	0.97	0.94	17.27	0.10

AUC = area under the curve, PPV = positive predictive value, NPV = negative predictive value, PLR = positive likelihood ratio, NLR = negative likelihood ratio, − = not applicable, ∞ = infinity.

**Table 2:** The posterior test probabilities of scoliosis-positive and negative screen.

Asymmetry index	Expected Cobb angle	One-time (%)	Two-time (%)	Three-time (%)
≤1.268	≤10°	0.14	0.006	0.0002
1.268≤	10°≤	33.0	86.9	98.9

## Discussion

3D depth sensors have been indicated for the screening of scoliosis^16,17^. Komeili *et al*.^16,17^ developed a 3D markerless method to investigate torso asymmetry and identified characteristic patters of asymmetry in AIS patients. They used four high-resolution laser scanners to be placed in the corners of a square room, and the subjects must stand in a custom-made frame to standardize the position relative to the scanners. The four views from the sensors must be merged, and its results strictly rely on the initial position. Conversely, our system does not require a strict position. In addition, a single widely available sensor is used for data acquisition that has the advantage to avoid calibration issues.

In this study, the correlation coefficient between Cobb angle and asymmetry index was 0.85, which exceeds the reported value for Adam’s forward bend test using a scoliometer (0.677)^8^. When predicting AIS of >10°, the ROC was 0.98, sensitivity was 0.97, and specificity was 0.93, indicating that this system has outstanding discriminative ability for deciding whether an examinee needed an additional radiographic examination to confirm the diagnosis of scoliosis. These results were in the same range as those in the previous pilot method validation study^2^. In addition, the posterior test probability for a positive screen >10° was 98.9% if the asymmetry index was >1.268 three times in a row. When a subject is suspected of having AIS (Cobb angle >10°) using this system (asymmetry index >1.268), the subject receives two additional tests, which eliminate the need for radiography when diagnosing AIS.

Mass school scoliosis screening programs for AIS were started in the 1950s^18^. However, the usefulness of school scoliosis screening stays controversial owing to its false positive referral rate and excessive costs^2,18,19^. In 2004, the United States Preventative Services Task Force advocated against routine screening of asymptomatic adolescents for AIS^2,20,21^. This may have been because there was no innovative, cost-effective screening tool available at the time. Nevertheless, the American Academy of Orthopaedic Surgeons (AAOS), the Scoliosis Research Society (SRS), the Pediatric Orthopaedic Society of North America (POSNA), and the American Academy of Pediatrics (AAP) released a position statement evincing the importance of such a program^22^. In addition, these same organizations stated that effective screening programs must have well-trained screening personnel who can utilize forward bend tests and scoliometer measurements to correctly identify and appropriately refer individuals with AIS for further investigation^22^.

Trunk asymmetry does not significantly correlate with scoliosis^23,24^. Therefore, evaluating the body surface in the standing posture is obviously limited in its ability to detect of scoliosis because the discriminative hump is not prominent in this posture^2^. The Adam’s forward bend test seeks to differentiate between faulty posture and actual AIS^2,21^; therefore, the scoliometer, used in the Adam’s forward bend test, is the best instrument available for scoliosis screening^2,21,22^. Indeed, there might not be a constant correlation between rotation of the trunk and angular deformities. Some patients have mild scoliosis and moderate rotation while others have significant scoliosis with mild trunk rotation.

Rotation is certainly the key element being examined but serves only as a guideline indicating the need for further evaluation. Nonetheless, our newly developed system also requires forward bend tests and automatically calculates an asymmetry index, in addition to a deviation contour map, indicating that it can be used in place of the traditional forward bend test and scoliometer measurements.

Children with tight hamstring muscles may not bend forward symmetrically. Some bend forward with one knee flexed, giving the illusion of trunk asymmetry. However, in the current system, even if the original data obtained from the patient’s back show left or right tilt, utilizing the best fit processing shows rotation of the reflected data, maintaining the best fit in reference to the original data^2^. Accordingly, the position of the reflected data, which institutes the basis for calculation of the asymmetry, depends mostly on the original data, which are unambiguously determined^2^. Therefore, the results are mostly unaffected by the patient’s posture or orientation^2^.

Because our previous study regarding repeatability analysis and the effects of trunk rotation (5° clockwise and counterclockwise rotations) already revealed that the coefficient of variation for each phantom model after 10 trials was 1–4%, which is regarded as very good repeatability^2^, intra correlation coefficients were not computed in this clinical study.

One of the strengths of the current study was its multicenter design. The fact that the system could be used at different institutions proved that it could be generalized. Conversely, one important limitation to this study relates to our subjects. This cohort included only patients that were referred to the study center with suspected AIS. This presents a possible selection bias as only 4/170 had straight spines (no scoliosis). While this is fine when determining correlation between Cobb measurements and the asymmetry index, it may overestimate the true sensitivity and specificity when used as a screening tool in a primary care clinic or in a school where most students have no scoliosis. Additional data is ultimately needed to determine if this tool is truly more sensitive and specific than physical exam and scoliometer measures. Such studies would provide a more robust proof-of-concept trial for the device. Therefore, further large-scale clinical trials targeting mass school scoliosis screening programs are needed. In addition, we are currently comparing our approach with other machine learning approaches such as neural networks and regression approaches that use point clouds and X-rays. Importantly, our approach demonstrates outstanding discriminative ability. However, machine learning or regression approaches may be useful for determining the need for reassessments in cases where the subject’s back is inadequately captured.

In conclusion, the novel 3D depth sensor imaging system is objective and less labor-intensive than traditional scoliosis screening methods and has outstanding discriminative ability for identification of idiopathic scoliosis in children and adolescents. These findings should be confirmed in studies featuring larger study cohorts that involve mass school scoliosis screening programs, possibly in the form of a multicenter trial.

## Data Availability

The data that support the findings of this study are available from the corresponding author on reasonable request.
